# Glycosylation and S-palmitoylation regulate SARS-CoV-2 spike protein intracellular trafficking

**DOI:** 10.1016/j.isci.2022.104709

**Published:** 2022-07-03

**Authors:** Chih-Feng Tien, Wan-Ting Tsai, Chun Hwa Chen, Hui-Ju Chou, Mingzi M. Zhang, Jhe-Jhih Lin, En-Ju Lin, Shih-Syong Dai, Yueh-Hsin Ping, Chia-Yi Yu, Yi-Ping Kuo, Wei-Hsiang Tsai, Hsin-Wei Chen, Guann-Yi Yu

**Affiliations:** 1 National Institute of Infectious Diseases and Vaccinology, National Health Research Institutes, Zhunan, Taiwan; 2 Institute of Molecular and Genomic Medicine, National Health Research Institutes, Zhunan, Taiwan; 3 Department and Institute of Pharmacology, School of Medicine, National Yang Ming Chiao Tung University, Taipei, Taiwan; 4 Graduate Institute of Biomedical Sciences, China Medical University, Taichung, Taiwan; 5 Graduate Institute of Medicine, College of Medicine, Kaohsiung Medical University, Kaohsiung, Taiwan

**Keywords:** Biochemistry, Virology, Cell biology

## Abstract

Post-translational modifications (PTMs), such as glycosylation and palmitoylation, are critical to protein folding, stability, intracellular trafficking, and function. Understanding regulation of PTMs of SARS-CoV-2 spike (S) protein could help the therapeutic drug design. Herein, the VSV vector was used to produce SARS-CoV-2 S pseudoviruses to examine the roles of the ^611^LYQD^614^ and cysteine-rich motifs in S protein maturation and virus infectivity. Our results show that ^611^LY^612^ mutation alters S protein intracellular trafficking and reduces cell surface expression level. It also changes S protein glycosylation pattern and decreases pseudovirus infectivity. The S protein contains four cysteine-rich clusters with clusters I and II as the main palmitoylation sites. Mutations of clusters I and II disrupt S protein trafficking from ER-to-Golgi, suppress pseudovirus production, and reduce spike-mediated membrane fusion activity. Taken together, glycosylation and palmitoylation orchestrate the S protein maturation processing and are critical for S protein-mediated membrane fusion and infection.

## Highlights

• ^611^LY^612^ mutation alters the glycosylation pattern of the SARS-CoV-2 S protein
• ^611^LY^612^ mutation reduces S protein surface expression level
• Palmitoylation targets mature S protein to the Golgi and plasma membrane
• Palmitoylation is required for pseudovirus and SARS-CoV-2 production

## Graphical abstract

**Figure:**
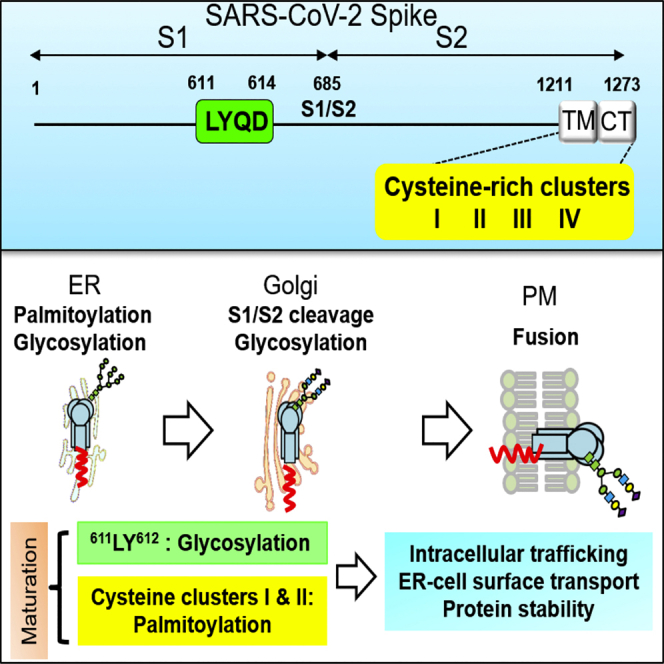

## Introduction

Severe acute respiratory syndrome coronavirus 2 (SARS-CoV-2), emerged in Wuhan, China, in December 2019 and caused a coronavirus disease (COVID-19) outbreak. SARS-CoV-2 belongs to Betacoronavirus, which contains Middle East respiratory syndrome coronavirus (MERS-CoV), SARS-CoV-1, and mouse hepatitis virus (MHV) and has a high similarity to SARS-CoV-1 (Korner et al., 2020; Woo et al., 2010). The SARS-CoV-2 spike (S) glycoprotein interacts with angiotensin-converting enzyme 2 (ACE2) on the cell surface during virus entry (Hoffmann et al., 2020b) to mediate virus-host membrane fusion (Huang et al., 2020). After translation, the coronavirus S protein is processed by host proteases into S1 and S2 subunits, and the S2 protein is further cleaved at the S2′ site to facilitate virus entry (Peng et al., 2021). The SARS-CoV-1 and SARS-CoV-2 S proteins have ∼76% amino acid identity (Lan et al., 2020; Zhou et al., 2020). In the SARS-CoV-2 S protein, an additional furin-like recognition sequence (RRAR^685^↓S) is present at the S1/S2 cleavage site, which may contribute to the high transmissibility of SARS-CoV-2 (Coutard et al., 2020; Hoffmann et al., 2020a; Papa et al., 2021; Peacock et al., 2021; Xia et al., 2020).

The S glycoprotein is a critical target for pathogenic coronavirus vaccine development, and current COVID-19 vaccines employ full-length or portions of S protein as the antigen to induce neutralizing antibodies against SARS-CoV-2 entry (Li et al., 2020; Tregoning et al., 2020). A process by which suboptimal antibodies against viral glycoproteins enhance viral infection through the Fcγ receptor (Lee et al., 2020), antibody-dependent enhancement (ADE) has been a concern in vaccine development against pathogens such as dengue virus (Katzelnick et al., 2017; Ulrich et al., 2020), SARS-CoV-1, and MERS-CoV (Wan et al., 2020; Wang et al., 2016). Several studies show that ADE of SARS-CoV-2 is mediated by Fcγ receptor IIA or complement component C1q (Maemura et al., 2021; Okuya et al., 2022; Wang et al., 2022). Monoclonal antibodies specific for the ^597^LYQD^600^ motif of the SARS-CoV-1 S protein are shown to have ADE activity (Wang et al., 2016). An LYQD motif is also present in the SARS-CoV-2 S protein, but whether eliminating the ADE-associated sequence in the S antigen is beneficial for an effective COVID-19 vaccine design remains unknown.

Maturation of the S glycoprotein is critical for coronavirus infection and transmission and can also be one of the antiviral targets. The S protein undergoes several post-translational modifications (PTMs), including N-linked glycosylation, palmitoylation, and proteolytic processing as part of its maturation process (Fung and Liu, 2018). 12 out of 23 asparagine residues in the SARS-CoV-1 S protein are glycosylated (Krokhin et al., 2003). SARS-CoV-1 S proteins are glycosylated in the endoplasmic reticulum (ER) with high-mannose glycans, which are then further modified as complex N-glycans in the Golgi (Duan et al., 2020; Nal et al., 2005). Glycosylation can influence viral glycoprotein folding, function, immune evasion, and virus infection (Huang et al., 2021; Watanabe et al., 2019, 2020). The endodomains of SARS-CoV-1 and SARS-CoV-2 S proteins contain a cysteine-rich motif for palmitoylation, which might participate in membrane fusion and infectivity (Petit et al., 2007; Wu et al., 2021). It has been shown that zinc finger DHHC domain palmitoyltransferase 5 (zDHHC5) and Golgin subfamily A membrane 7 (GOLGA7) interact with S protein and induce its palmitoylation (Gordon et al., 2020; Wu et al., 2021; Zeng et al., 2021). How these PTMs affect SARS-CoV-2 S protein stability, intracellular trafficking, and function will need to be addressed more thoroughly.

In this study, the vesicular stomatitis virus (VSV) vector was used to generate the SARS-CoV-2 S pseudoviruses for the functional characterization of the LYQD and cysteine-rich motifs on S protein maturation and virus infectivity. Our results suggest that the LYQD motif was involved in the S protein glycosylation process, and the palmitoylation of the cysteine-rich motif participated in the S protein trafficking and maturation process.

## Results

### Mutations in the LYQD motif change the glycosylation pattern of the SARS-CoV-2 S protein

**Figure 1:**
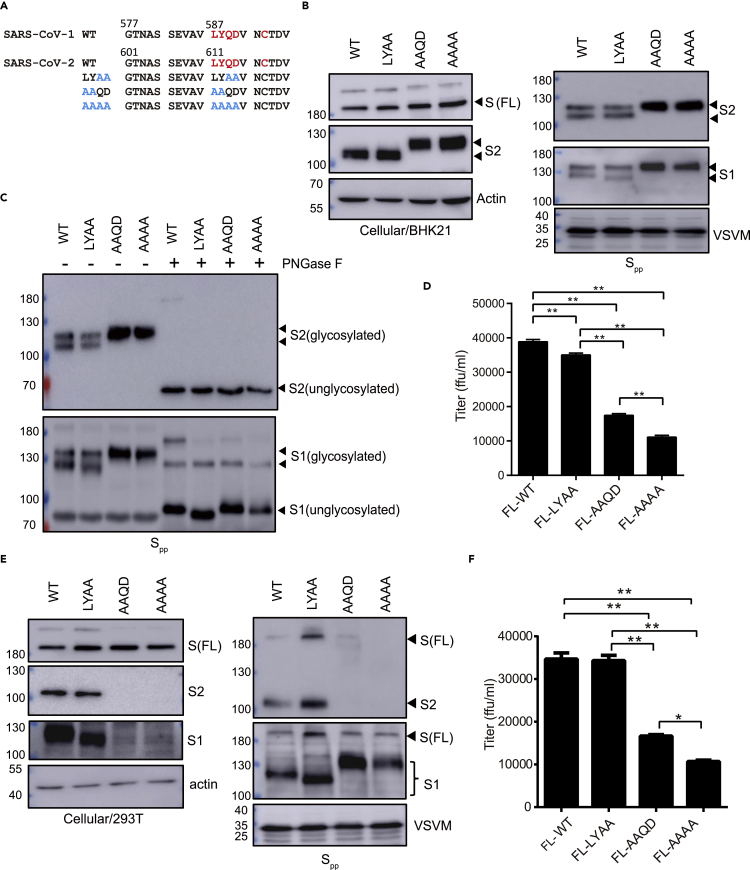
The ^611^LY^612^ mutation in the antibody-dependent enhancement (ADE) domain leads to aberrant glycosylation of the SARS-CoV-2 spike (S) protein (A) Amino acid sequences of the wild type (WT) and mutants close to the putative ADE domain of the SARS-CoV-2 spike protein. Red: ADE domain; Blue: mutation sites. (B) BHK21 cells were transfected with plasmid DNA to introduce full-length (FL) spike protein expression and then infected with the recombinant VSV defective in glycoprotein expression (VSVΔG-GFP/G) for pseudovirus production. Spike protein expression in cells at 24 h post-transfection and in S pseudovirus (S_pp_) in the culture supernatant at 16 h post-VSVΔG-GFP/G infection was examined by immunoblotting. (C) Assessment of S protein glycosylation in S_pp_ via immunoblot after treatment with peptide N-glycosidase F (PNGase F). (D) Quantification of the S_pp_ titer via counting the GFP-positive BHK21-hACE2 cells at 16 h post-infection. (E and F) 293T cells were used to express the LYQD-related S mutant protein and generate the mutant S_pp_. The protein expression in cell and in S_pp_ was detected by immunoblotting (E), and the S_pp_ titer was measured in BHK21-hACE2 cells (F). ∗∗p < 0.01. Error bars represent SEM and n = 3.

Antibodies recognizing the ^587^LYQD^590^ and C^593^ amino acid residues in the SARS-CoV-1 S protein have ADE activity (Wang et al., 2016). To examine whether the ^611^LYQD^614^ sequence in SARS-CoV-2 S protein maturation can be removed from the S protein, alanine substitution mutations in the LYQD sequence (^611^LYAA^614^, ^611^AAQD^614^, and ^611^AAAA^614^) were generated in the mammalian expression plasmid (Figure 1A). As the following cysteine residue might be involved in inter- or intra-molecule disulfide bond formation, C^617^ of SARS-CoV-2 S protein was not mutated. Wild type (WT) and mutant S protein expression in BHK21 cells was achieved through plasmid DNA transfection, followed by infection with the VSVΔG-GFP/G virus to secrete the S pseudotyped virus particles (S_pp_) into the culture medium. When the S protein expression in transfected cells was examined by immunoblotting with an anti-S2 antibody, full-length S protein (∼180 kDa) and S2 (∼100 kDa) were both detected in the cell lysate (Figure 1B, left panel). However, the S2 proteins of the AAQD and AAAA mutants had different electrophoretic mobility in contrast to those from the WT and LYAA mutants. S_pp_ secreted into the culture supernatant was then examined by immunoblotting with antibodies specific for the S1 and S2 proteins (Figure 1B, right panel). Very little unprocessed FL S protein was detected in the S_pp_ culture medium. The S1 and S2 proteins from WT and LYAA S_pp_ each appeared as a doublet on an immunoblot, indicating two different protein sizes, but appeared as a single high molecular weight band in the culture medium containing AAQD and AAAA S_pp_.

Because the SARS-CoV-2 S protein has 22 predicted N-linked glycosylation sites, with the N^603^ and N^616^ residues located close to the LYQD motif (Watanabe et al., 2020), we postulated that the differential electrophoretic mobility observed for the S1 and S2 mutants could be due to changes in protein glycosylation. To test this, WT and mutant S_pp_ were treated with peptide N-glycosidase F (PNGase F) to remove N-linked oligosaccharides from the S protein. The protein size of S1 and S2 proteins from WT, AAQD, and AAAA S_pp_ became the same after PNGase F treatment (Figure 1C), suggesting that the ^611^LY^612^ mutation altered the glycosylation pattern of S protein. When the S_pp_ titer was measured by assessing the GFP signal in infected BHK21-hACE2 cells, the AAQD and AAAA S_pp_ had significantly lower viral titer compared to the WT and LYAA S_pp_ (Figure 1D). Taken together, these data suggest that mutation of LY residues affects the glycosylation pattern of S protein.

### ^611^LY^612^ sequence is required for high titer of S_pp_ production in HEK293T

To test whether the LYQD sequence is also required for S_pp_ production in other cell types, HEK293T was used for S_pp_ packaging (Figures 1E and 1F). While the WT and LYAA mutant proteins show normal S protein processing and S_pp_ production, only one dominant S1 and S2 form were present in S_pp_ from HEK293T (Figure 1E), which was different from the S_pp_ generated from BHK-21 cells. The S2 subunit of the AAQD and AAAA mutants might be unstable and could not be detected in HEK293T cell and medium. Surprisingly, a large amount of S1 subunit was detected in the culture medium. When the virus particles were precipitated with PEG (Figure S1), S1 and S2 subunits were present in the WT and LYAA S_pp_ but not in the AAQD and AAAA S_pp._ This suggests that the S1 subunit in AAQD and AAAA mutants was present in the culture medium but not associated with virus particles. Viral titers of AAQD and AAAA S_pp_ were also consistently reduced (Figure 1F).

The SARS-CoV-2 S protein contains 22 asparagine (N) residues, 16 of which have N-linked glycosylation (Shajahan et al., 2020; Walls et al., 2020; Watanabe et al., 2020). The N^603^ and N^616^ residues are close to ^611^LYQD^614^ sequence. Whether N^603^ and N^616^ mutation to alanine in S protein might show a similar phenotype as LY mutation was further tested. As shown in Figure S2A, the protein expression level and glycosylation pattern of the N603A and N616A mutants were very similar to the WT protein, suggesting that the phenotype of the ^611^LY^612^ mutation is not derived from the dysregulation of the neighboring glycosylation sites. Interestingly, the infectivity of the N603A and N616A S_pp_ was significantly reduced (Figure S2B), indicating that appropriate glycosylation is critical for S protein-mediated infection.

The C-terminal tail of the S protein contains an ER-retention motif, and deletion of the tail facilitates S protein targeting to the plasma membrane for pseudovirus production (Lontok et al., 2004; McBride et al., 2007; Xiong et al., 2020). The S mutants with truncation of the C-terminal 19 amino acids (SΔ19) and LYQD-related mutations were generated to evaluate S_pp_ production efficiency. The WT and LYAA SΔ19_pp_ were produced with high efficiency, as indicated by immunoblotting and virus titration (Figure S3). The AAQD and AAAA SΔ19_pp_ production were much lower than the WT and LYAA SΔ19_pp_. These data indicate that the LY sequence was required for a high titer of S_pp_ and SΔ19_pp_ production.

### ^611^LY^612^ sequence is required for S protein maturation and trafficking

**Figure 2:**
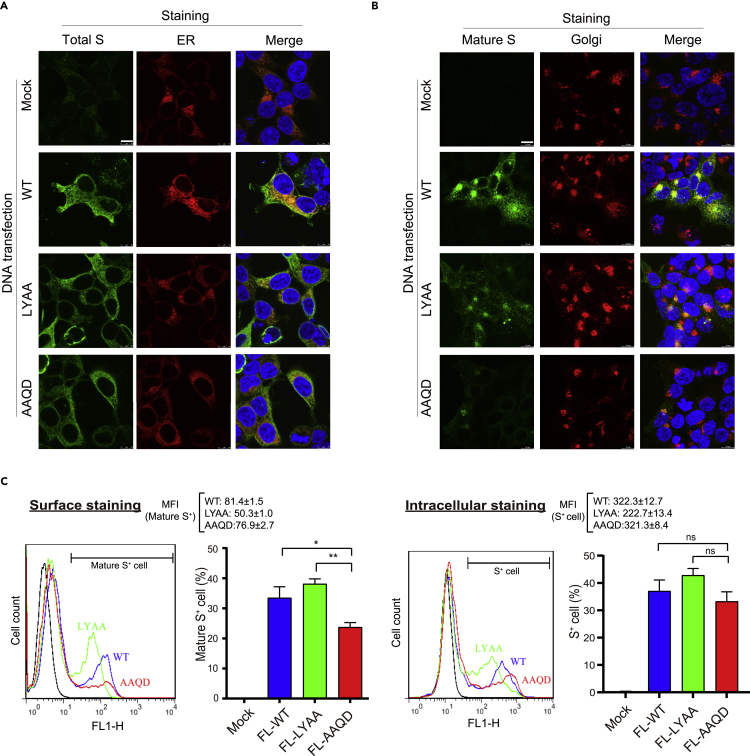
^611^LY^612^ mutation blocks S protein maturation (A and B) 293T/17 cells were transfected with plasmid DNA encoding WT and mutant S proteins, and the subcellular expression pattern was examined by immunofluorescent staining with the antibodies specific for S2 (1A9 and ECD45 clones), PDI (ER marker), and RCAS1 (Golgi marker). The nascent S protein was recognized by an anti-S2 (1A9) antibody (A). Mature S protein was stained with an anti-S2 (ECD45) antibody (B). Scale bars: 10 μm. (C) The 293T/17 cells expressing S protein were subjected to surface staining with anti-S2 (ECD45) antibody and intracellular staining with anti-S2 (1A9) antibody for flow cytometry. MFI: mean fluorescence intensity of the positive cells. ∗p < 0.05, ∗∗p <0.01. Error bars represent SEM and n=3.

Protein glycosylation with diverse glycan occurs in the ER and Golgi. As ^611^LY^612^ mutation altered the glycosylation profile on both S1 and S2 subunits, we next examined whether ^611^LY^612^ sequence affects S protein subcellular distribution. When the expression pattern of S protein was examined by immunostaining with antibodies recognizing the S2 epitope (1A9), WT, LYAA, and AAQD S proteins were present in the cytoplasm and partially colocalized with the ER marker, which might correlate with the nascent S protein localization (Figure 2A). When an anti-S2 antibody (ECD45) with a high affinity to the prefusion state of trimeric S (mature S) was used for immunostaining, the mature WT and LYAA S protein expression were concentrated in the Golgi, and some speckles in the cytoplasm (Figure 2B). LY mutation showed a slightly reduced mature S expression compared to WT by immunostaining. In contrast, the signals for mature S of the AAQD mutant were very weak, and rarely present in the Golgi, suggesting that the LY mutation downregulates the S protein trafficking and maturation process. Some S proteins might further transport from Golgi to the cell surface, which may contribute to cell-cell fusion and spreading of SARS-CoV-2. Hence, whether the LYQD mutation affects S protein expression on the cell surface with anti-S2 antibodies (1A9 and ECD45) was examined by flow cytometry. As shown in Figure 2C, a higher percentage of cells had mature S protein expression on the cell surface in the WT and LYAA plasmid-transfected cells than in the AAQD-transfected group. The percentages of intracellular S protein expression of the WT, LYAA, and AAQD plasmid-transfected cells did not show any significant difference. The mean fluorescence intensity (MFI) of the LYAA mutant on the cell surface or intracellular was lower compared to the WT, suggesting that the protein stability of the LYAA mutant might also be slightly affected. Taken together, the LYQD sequence might sustain S protein stability and facilitate S protein moving from ER to Golgi for glycan modification and further to the plasma membrane during its maturation.

### Palmitoylation is required for efficient S_pp_ and SARS-CoV-2 production

**Figure 3:**
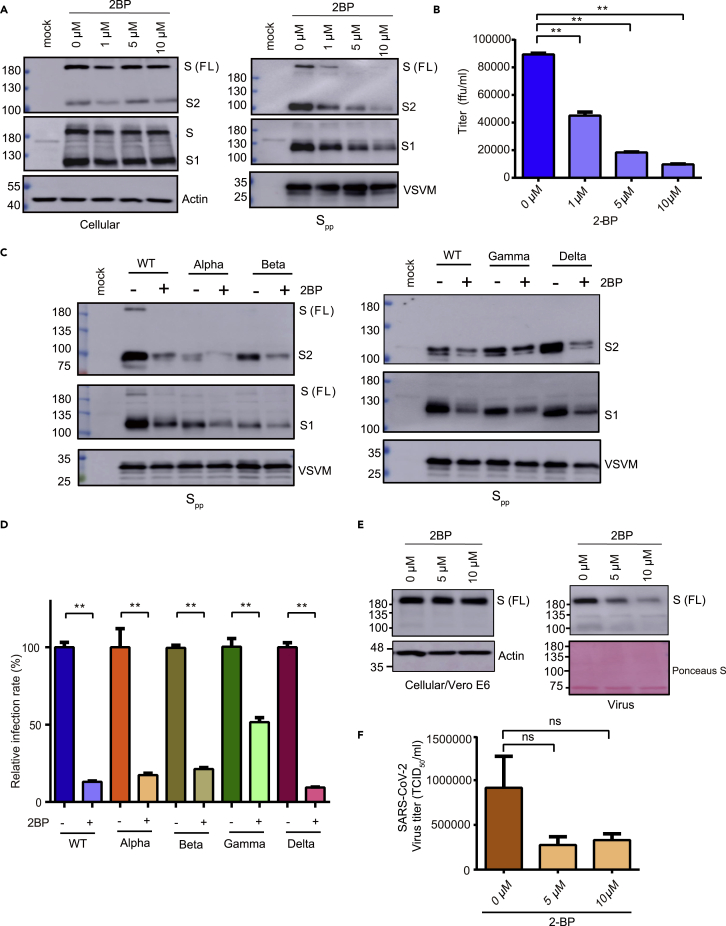
2-Bromopalmitate (2BP) inhibits S_pp_ and SARS-CoV-2 production (A) Expression of S protein in 293T/17 cells and S_pp_ in the presence of 2BP treatment (1–10 μM) were examined by immunoblotting. (B) The S_pp_ titer was evaluated in BHK21-hACE2 cells. (C) The WT and variant (alpha, beta, gamma, and delta strains) S_pp_ generated from 293T/17 cells in the presence of 2BP treatment (10 μM) was examined by immunoblotting. (D) The WT and variant S_pp_ titers were evaluated. (E and F) Vero E6 cells were infected by SARS-CoV-2 (hCoV-19/Taiwan/4/2020, moi = 1) in the presence of 2BP. The cell lysate and supernatant were collected at 48 h post-infection and subjected to immunoblotting (E) and virus titration (F). ∗∗p < 0.01. Error bars represent SEM and n = 3.

Protein palmitoylation primarily functions in protein subcellular trafficking. The endodomain of SARS-CoV-2 S protein contains a cysteine-rich motif for palmitoylation. To evaluate the function of palmitoylation in S protein maturation and virus infectivity, full-length S protein expression and S_pp_ packaging in HEK293T cells were examined in the presence of a general palmitoylation inhibitor, 2-bromopalmitate (2BP). While S, S1, and S2 protein intracellular expression was not altered by the 2BP treatment at non-toxic concentrations (1–10 μM; Figures 3A and S4), the S_pp_ packaging and titer in the culture medium were reduced (Figures 3A and 3B). As the cysteine-rich motif remains intact in the SΔ19 protein, the SΔ19_pp_ production was also inhibited by the 2BP treatment (Figure S5). Genetic lineages of SARS-CoV-2 continue to evolve some variants, such as alpha, beta, gamma, and delta variants, which may have a higher transmission rate and decrease the effectiveness of COVID-19 vaccines (Chakraborty et al., 2021). Palmitoylation inhibition by 2BP reduced variant S_pp_ packaging and virus titer (Figures 3C and 3D) but did not affect the S, S1, and S2 intracellular expression (Figure S6). To examine the importance of S protein palmitoylation in SARS-CoV-2 virus replication, Vero E6 cells were infected with SARS-CoV-2 virus (moi = 1) in the presence of 2BP treatment for 48 h. As shown in Figure 3E, palmitoylation inhibition did not affect intracellular S protein expression but blocked SARS-CoV-2 virus production in the culture medium. The virus titer was slightly lower in the presence of 2BP (Figure 3F). In summary, palmitoylation inhibition suppresses S_pp_ and SARS-CoV-2 virus production.

### SARS-CoV-2 S protein is palmitoylated at multiple cysteine residues

**Figure 4:**
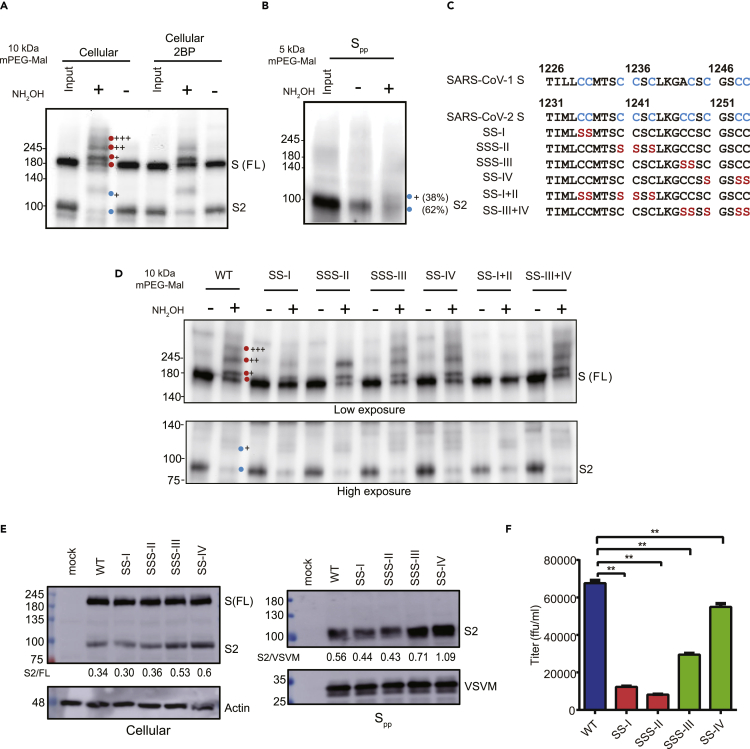
Palmitoylation of WT and cysteine-rich cluster mutants S protein (A) Palmitoylated proteins in 293T/17 cells were treated with 10 kDa methoxy polyethylene glycol maleimide (mPEG-mal) and immunoblotted with an anti-S2 antibody. (B) Palmitoylated S protein in S_pp_ was labeled with 5 kDa mPEG-mal and detected by immunoblotting. (C) Sequence of cysteine-rich motif of the WT and mutant S proteins. (D) Palmitoylation of cysteine-rich cluster mutants as determined by acyl-PEG exchange. (E) The S protein expression of WT and cysteine-rich cluster mutants in 293T/17 cells and S_pp_ was examined by immunoblotting with an anti-S2 antibody. (F) The titers for WT and cysteine mutants S_pp_ were evaluated. Error bars represent SEM and n = 3. Red circle: S protein; blue circle: S2 subunit. +: number of different palmitoylated species.

To confirm S protein palmitoylation was inhibited by 2BP, the palmitoylated S protein was monitored by using a modified protocol of the acyl-PEG exchange gel (Percher et al., 2016). As shown in Figure 4A, there were at least three palmitoylated species of the S protein as observed by the slower migrating bands on the Western blot after acyl-PEG exchange. The palmitoylation level of S protein was inhibited by the 2BP treatment. We also detected a single major palmitoylated species of the S2 protein with or without 2BP treatment. Similarly, the palmitoylation level of SΔ19 was also suppressed by the 2BP treatment (Figure S7). Furthermore, the palmitoylated S2 protein was also incorporated in the S_pp_ virus (Figure 4B), suggesting that S protein palmitoylation might directly participate in virus packaging.

The cysteine-rich motifs of SARS-CoV-1 and SARS-CoV-2 S protein each contain four clusters known to be involved in protein palmitoylation. To examine the functional importance of the cysteine-rich clusters in SARS-CoV-2 S protein palmitoylation and S_pp_ production, we generated cysteine-to-serine mutations in the cysteine-rich motif of S protein (SS-I, SSS-II, SSS-III, SS-IV, SS-I + II, and SS-III + IV) (Figure 4C). Consistent with previous reports (Petit et al., 2007; Wu et al., 2021), clusters I and II are crucial for SARS-CoV-2 S protein palmitoylation. SS-I and SSS-II mutants showed drastically diminished palmitoylation levels and palmitoylated forms S protein were barely detectable for the SS-I + II mutant (Figure 4D). In contrast, the major palmitoylated species of the SARS-CoV-2 S protein were still observed for the SSS-III, SS-IV, and SS-III + IV mutants. S protein palmitoylation is important for S_pp_ production because S2 expression level in S_pp_ and S_pp_ titers was reduced in the SS-I, SSS-II, and SS-I + II mutants (Figures 4E, 4F, and S8). Interestingly, the SSS-III, SS-IV, and SS-III + IV mutants showed higher S_pp_ production but reduced virus titers (Figures 4E and S8), suggesting that cysteine clusters III and IV might have additional roles in S protein processing and S_pp_ production that are independent of protein palmitoylation. Collectively, these results demonstrate that cysteine clusters I and II are important for S protein palmitoylation and S_pp_ production.

### Palmitoylation targets mature S protein to the Golgi and plasma membrane

**Figure 5:**
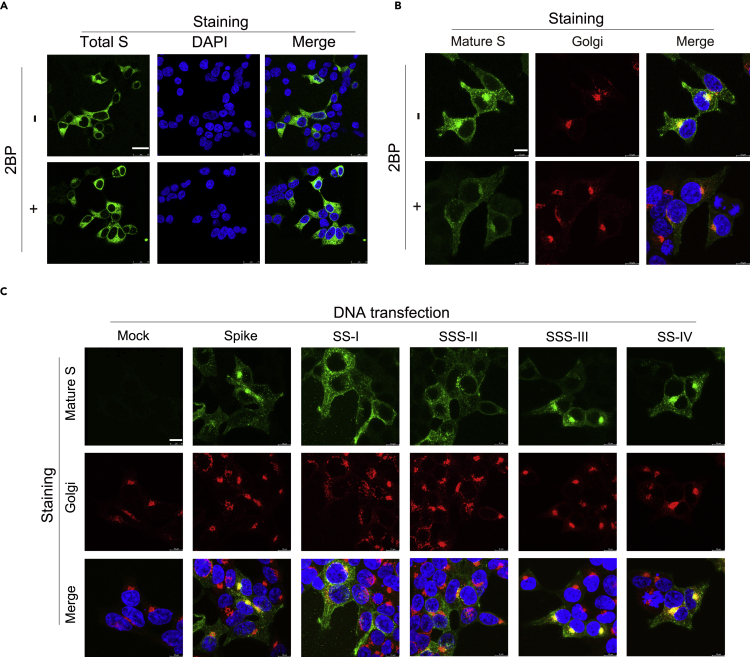
Palmitoylation inhibition and cysteine-rich clusters I & II mutation blocks S protein maturation (A and B) The mature S protein in the DNA transfected 293T/17 cells with or without 2BP treatment for 24 h was fixed and stained with antibodies specific for (A) S1 and (B) S2 & RCAS1 (Golgi marker). Scale bars: 25 μm (A) or 10 μm (B). (C) The S protein carrying cysteine-rich cluster mutations in 293T/17 cells (24 h) were fixed and stained with antibodies specific for S2 and RCAS1 (Golgi marker). Nuclear DNA was counterstained with DAPI dye. Scale bars: 10 μm.

As palmitoylation may influence protein localization, trafficking, and stability (Aicart-Ramos et al., 2011; Yang et al., 2020), we evaluated whether palmitoylation modulates S protein subcellular localization. The localization of nascent S protein was unaffected with 2BP treatment when the S protein was stained with anti-S1 antibody (clone HL263) (Figure 5A). In contrast, the localization of mature S protein stained by ECD45 antibody was concentrated in the Golgi, plasma membrane, and speckles in the cytoplasm. Upon 2BP treatment, the fluorescent intensity of mature S protein was reduced and dispersed as an ER-like distribution (Figure 5B). The S protein subcellular localization of the cysteine cluster mutants was also examined. Similarly, consistent with palmitoylation targeting mature S protein to the Golgi, the palmitoylation-defective SS-I and SSS-II mutant S proteins were distributed outside the Golgi (Figure 5C). On the other hand, the mature SSS-III and SS-IV S proteins remained predominantly concentrated in the Golgi and plasma membrane. Furthermore, the mature SS-I + II mutant showed a more dispersed compartment outside the Golgi, and the SS-III + IV combination mutant was even more condensed in the Golgi and plasma membrane despite similar localization of the nascent S proteins (Figure S9). In summary, palmitoylation is important for the subcellular localization and trafficking of the S protein.

### S protein palmitoylation is required for the ACE2-mediated cell fusion

**Figure 6:**
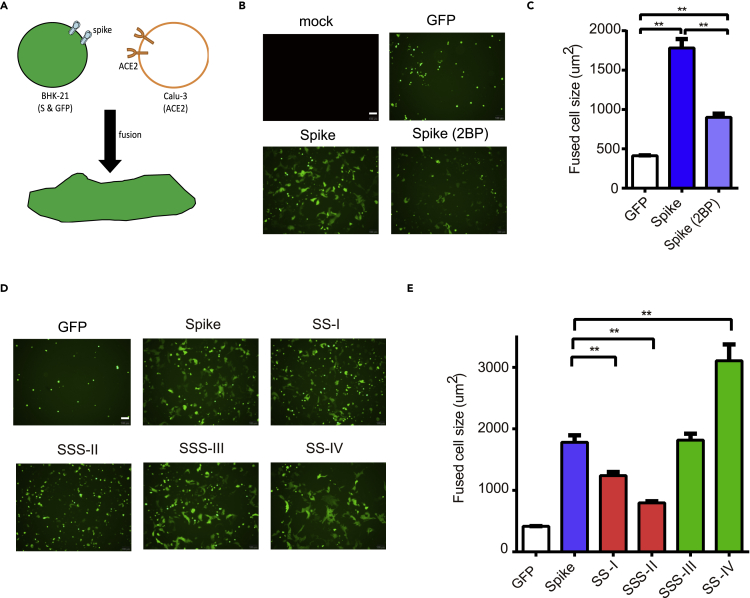
Palmitoylation of S protein facilitates the ACE2-mediated cell fusion (A) S protein-ACE2-mediated cell fusion assay. (B and C) The S and GFP coexpression plasmids were introduced to BHK21 cells by DNA transfection for 24 h. The transfected cells were collected and overlaid on Calu-3 cells at 4°C for 1 h, and the unbound cells were removed with PBS wash. After further 4 h incubation, the images of the GFP^+^ cells were captured by fluorescent microscope. GFP^+^ cell sizes in five randomly selected fields were quantified using ImageJ (C). (D and E) The cysteine mutants were subjected to the cell fusion assays (D). GFP^+^ cell sizes in five randomly selected fields were quantified using ImageJ (E). Scale bars: 100 μm ∗∗p < 0.01. Error bars represent SEM and n = 3.

Other than assembling into the virion, mature S proteins may be expressed on host cell surfaces and induce membrane fusion with neighboring cells (Duan et al., 2020; Nishima and Kulik, 2021). As some palmitoylated S proteins observed at the plasma membrane (Figure 5), we examined whether palmitoylation is required for S protein-mediated cell fusion. GFP- and S-expressing BHK-21 cells were overlaid on the calu-3 cell film at 4°C for 45 min. After washing with PBS, the S protein-ACE2-mediated cell fusion between BHK-21 and Calu3 was monitored by tracing the GFP^+^ multinucleated cells after 4 h incubation (Figure 6A). The GFP^+^ cells without S protein expression remained as single cells (300–400 μm^2^) while those with S protein expression became large multinucleate cells (1500–2000 μm^2^) upon cell fusion (Figures 6B and 6C). Upon palmitoylation inhibition by 2BP, the GFP^+^ multinucleate cells had reduced cell-cell fusion (1000 μm^2^ on average). Consistent with S palmitoylation being important for spike-mediated membrane fusion, significantly reduced cell fusion was observed for palmitoylation-defective SS-I and SSS-II mutants (Figures 6D and 6E). Further highlighting the different functions of clusters III and IV, cell-cell fusion was not reduced when these clusters were mutated. Interestingly, SS-IV mutation enhanced the cell-cell fusion by about 50%, although likely through palmitoylation-independent mechanisms. Taken together, these data indicate that the palmitoylation of S protein in cysteine clusters I and II is involved in the mature S protein trafficking to the Golgi and cell surface to facilitate S-ACE2-mediated cell fusion.

## Discussion

The VSVΔG-based pseudovirus was applied to characterize SARS-CoV-2 S protein maturation and virus infection. The ^611^LY^612^ sequence in the putative ADE motif of S protein is required for correct glycosylation and maturation, which is essential for the production of infectious S_pp_ particles. In contrast, the S protein can tolerate ^613^QD^614^ mutation in the putative ADE motif without affecting glycosylation and virus infection. The ^613^QD^614^ could be further evaluated in a vaccination setting. Palmitoylation targets the mature S protein trafficking to the Golgi and plasma membrane, which subsequently facilitates pseudovirus packaging and cell-cell membrane fusion. The four cysteine clusters of S protein have distinct roles in these processes. The study reveals that glycosylation and palmitoylation coordinate the S protein maturation processes, which are critical for the protein to function.

Glycosylation on the newly translated protein facilitates correct protein folding. Glycans could influence the conformation of S protein (Sztain et al., 2021), affect the binding affinity between S protein and ACE2 receptor (Casalino et al., 2020; Koehler et al., 2020), and avoid S protein from antibody recognition (Wang, 2020; Watanabe et al., 2020; Zhao et al., 2021). Based on our results, the S protein in S_pp_ showed two different glycosylation forms in BHK21 cells but only one form (lower molecular weight) in 293T cells. The difference could be due to the diversity of glycosylation machinery in various cell types or fast degradation of the S protein with alternative glycosylation in 293T cells. The ^611^LY^612^ S protein with an altered glycosylation profile (higher molecular weight) could be incorporated in S_pp_ in BHK21 cells, and the ^611^LY^612^ mutant S_pp_ showed lower infectivity. The SARS-CoV-2 S protein contains multiple N-linked glycosylation sites, and N^603^ & N^616^ residues are close to ^611^LYQD^614^ sequence. The glycosylation levels of the N603A and N616A mutants are different from the ^611^LY^612^ mutant, suggesting that the low maturation process of the ^611^LY^612^ mutant is not due to the changes in the glycosylation of the neighboring amino acid sequence. Although the LY sequence is located in the S1 subunit, the S2 glycosylation profile was also affected, suggesting that the LY mutation keeps the S protein from trafficking to the Golgi, where glycan modification on both S1 and S2 subunits can be further processed. In 293T/17 cells, the S1 and S2 subunits of ^611^LY^612^ mutant might be unstable and subjected to protein degradation. It is also possible that the ^611^LY^612^ might impact protein folding, leading to ER quality control retention (Benyair et al., 2011).

Palmitoylation is important for SARS-CoV-1 and SARS-CoV-2 S protein maturation in order to function during virus infection and membrane fusion. Palmitoylation might facilitate S protein trafficking from ER to the cell surface during its maturation. The cysteine cluster I and II of SARS-CoV-1 and SARS-CoV-2 S proteins are the main palmitoylation site, as shown in our study and several studies (Mesquita et al., 2021; Petit et al., 2007; Puthenveetil et al., 2021). Host proteins zDHHC5 and GOLGA7 are involved in the palmitoylation of S protein (Gordon et al., 2020; Wu et al., 2021; Zeng et al., 2021). zDHHC enzymes such as zDHHC2, zDHHC3, zDHHC8, zDHHC9, and zDHHC20 may also contribute to the palmitoylation process of S protein (Mesquita et al., 2021; Puthenveetil et al., 2021; Ramadan et al., 2022). zDHHC enzymes distribute at ER, Golgi, and plasma membranes throughout the cellular secretory pathway (Malgapo and Linder, 2021). Zeng et al. showed that the intracellular distribution of S protein was not altered in the zDHHC5 or GOLGA7 knockout condition (Zeng et al., 2021), but our data show that mutations of the palmitoylation sites reduce the Golgi localization of the mature S protein. The discrepancy may be due to the difference in antibody selection for immunostaining. The other possibility was that the zDHHC family enzymes might compensate for the deficiency of the zDHHC5 or GOLGA7 and complete the palmitoylation of S protein. Mutations in cysteine clusters I and II also lead to a deficient ER-to-cell surface trafficking of the S protein, S1/S2 cleavage, virus packaging, and S-mediated membrane fusion. The cluster I and II mutants carry two palmitoylation sites of S proteins, whereas cluster III and IV mutants might contain more than two palmitoylation sites of S proteins. Moreover, the mutations in the cysteine clusters III and IV seem further enhance Golgi localization, S1/S2 cleavage, and virus packaging, suggesting that they might have a regulatory role in these processes. Based on the S protein structure prediction (Zheng et al., 2021), the cysteine-rich motif is located in the cytoplasmic tail proximally to the transmembrane domain. The cysteine-rich clusters I and II are in a stable alpha-helix structure and might be more accessible for zDHHC enzyme, which is known to interact with SARS-CoV-2 S protein. As the cysteine-rich motif in SARS-CoV-2 S is highly conserved in all the variants, S palmitoylation might be a potential target for antiviral drug development.

S protein trafficking is via the secretory pathway (Bracquemond and Muriaux, 2021; Mendonca et al., 2020). After synthesized, S protein is modified in ER and Golgi for post-translational modifications. It has been shown that the S protein has ER export and ER retrieval signals at the cytoplasmic tail (Cattin-Ortola et al., 2021). How the LYQD motif-associated glycosylation regulation and palmitoylation of S protein coordinate with these ER export and ER retrieval signals in the maturation process will be worth further investigation.

### Limitations of the study

First, we have utilized vesicular stomatitis virus pseudoviruses to study glycosylation and palmitoylation of S protein. However, it is difficult to develop SARS-CoV-2 infectious cDNA clone to understand glycosylation and palmitoylation during virus replication and package. Second, detecting ADE activity from AAQD and AAAA S_pp_ is not very easy because viral titers of AAQD and AAAA S_pp_ were lower than that of LYAA S_pp_. Further studies will be required to evaluate the glycosylation mechanisms of LYQD motif and palmitoylation of S protein coordinate with these ER signal peptides in the maturation process.

## STAR★Methods

### Key resources table

**Table:** 

REAGENT or RESOURCE	SOURCE	IDENTIFIER
**Antibodies**		

Anti-CoV-2 S2 (1A9)	Genetex, Hsinchu, Taiwan	GTX632604
Anti-VSV-M	Absolute, Boston, MA	Ab01404–2.0
Anti-beta-actin	Genetex, Hsinchu, Taiwan	GTX109639
Anti-CoV-2 Spike S1	Genetex, Hsinchu, Taiwan	GTX635672
Anti-CoV-2 mature S2 (ECD 45)	Antaimmu BioMed, Hsinchu, Taiwan	ATBM-A-08
Anti-PDI	Cell signaling, Danvers, MA	45596
Anti-RCAS1	Cell signaling, Danvers, MA	12290

**Bacterial and virus strains**		

DH5α	Yeastern Biotech, Taipei, Taiwan	FYE678
SARS-CoV-2	Taiwan Centers for Disease Control	N/A

**Chemicals, peptides, and recombinant proteins**		

PNGase F	NEB, Ipswich, MA	P0704S
Lipofectamine 2000	ThermoFisher, Waltham, MA	11668
DAPI	ThermoFisher, Waltham, MA	D21490
2-Bromohexadecanoic acid (2BP)	Sigma-Aldrich, Darmstadt, Germany	238422
M199 medium	Gibco; ThermoFisher, Waltham, MA	11150–059
TPCK-trypsin	Sigma-Aldrich, Darmstadt, Germany	T8802
MTT solution	Sigma-Aldrich, Darmstadt, Germany	M-0283
Versene solution	Gibco, Waltham, MA	15040066
Protease inhibitor cocktail	ThermoFisher, Waltham, MA	78437
Phenylmethylsulfonyl fluoride	Sigma-Aldrich, Darmstadt, Germany	329-98-6
Transcription Factor Buffer Set	BD Pharmingen™; ThermoFisher, Waltham, MA	AB_2869424
SuperNuclease	SinoBiological,Beijing,China	SSNP01
TCEP solution	ThermoFisher, Waltham, MA	77720
N-Ethylmaleimide	Sigma-Aldrich, Darmstadt, Germany	04259
mPEG-Mal	Sigma-Aldrich, Darmstadt, Germany	99126-64-4
MEM alpha medium	Cytiva; Hyclone, Marlborough, MA	SH30265
FBS	Cytiva; Hyclone, Marlborough, MA	SH30396
Pen Strep solution	Corning, New York	20-003
HEPES buffer	Biological Industries, Israel	03-025
DMEM/High glucose medium	Cytiva; Hyclone, Marlborough, MA	SH30243
MEM medium	ThermoFisher, Waltham, MA	11095080

**Experimental models: Cell lines**		

Vero E6	provided by Dr. Shiow-Ju Lee, NHRI	N/A
HEK293T/17	provided by Dr. Chia-Yi Yu, NHRI	N/A
BHK21	provided by Dr. Chia-Yi Yu, NHRI	N/A
Calu-3	provided by Dr. Yueh-Hsin Ping, NYCU	N/A

**Software and algorithms**		

GraphPad PRISM 6.2	https://www.graphpad.com	N/A
ImageJ	https://imagej.nih.gov/ij/	N/A

### Resource availability

#### Lead contact

Further information and requests for resources and reagents should be directed to and will be fulfilled by the lead contact, Guann-Yi Yu (guannyiy@nhri.edu.tw).

#### Materials availability

Plasmids in this paper will be shared by the lead contact upon request.

### Experimental model and subject details

#### Cell, virus, and reagent

BHK21 cells were maintained in MEM alpha medium (Cytiva; Hyclone, Marlborough, MA) containing 10% FBS (Cytiva; Hyclone, Marlborough, MA), 1× Penicillin Streptomycin (PS) solution (100 IU/mL Penicillin and 100 μg/mL Streptomycin, Corning, New York), and 1× HEPES buffer (Biological Industries, Israel). HEK293T/17 cells and Vero E6 cells were maintained in 10% FBS in DMEM/High glucose medium (Cytiva; Hyclone, Marlborough, MA) with 1× PS solution. Calu-3 cells were maintained in 10% FBS in MEM medium (ThermoFisher, Waltham, MA) with 1× PS solution. Human ACE2 (hACE2) overexpression in BHK21 cells was introduced by the lentiviral vector carrying the hACE2 gene as described previously (Su et al., 2020). SARS-CoV-2 virus (hCoV-19/Taiwan/4/2020) was kindly provided by the Taiwan Centers for Disease Control and was amplified in Vero E6 cells in M199 medium with 2 μg/mL TPCK-trypsin. All SARS-CoV-2 experiments were performed in a biosafety level 3 (BSL-3) laboratory. 2-Bromohexadecanoic acid (2BP) was purchased from Sigma-Aldrich (Darmstadt, Germany).

#### VSV based-SARS-CoV-2 S pseudovirus (S_pp_)

Full-length S (Wuhan-Hu-1 strain, MN908947.3) or C-terminal 19 amino acid deletion mutant (SΔ19) were PCR-amplified and inserted between *Nhe*I and *Not*I restriction enzyme sites in the pVax1 vector (ThermoFisher). Mutations of the ^611^LYQD^614^ sequence (LYAA, AAQD, and AAAA), N603A, and N616A were generated by site-directed mutagenesis. The VSVΔG-GFP/G virus was recovered from the rVSV-ΔG-GFP-2.6 plasmid and helper plasmids (G, L, N, and P; Kerafast, Boston, MA) using methods described previously (Hsu et al., 2021; Lin et al., 2022; Whitt, 2010). To generate VSV based-SARS-CoV-2 S pseudovirus (S_pp_) or related mutant viruses, BHK21 cells were transfected with pVax1-SARS-CoV-2 S or mutant plasmids using Lipofectamine 2000 (ThermoFisher, Waltham, MA) and infected with the VSVΔG-GFP/G virus (multiplicity of infection [moi] = 5) the next day. After incubation for 24 h, the supernatant was clarified by centrifugation at 1,320 g for 10 min and stored at −80°C. For titration, the BHK21-hACE2 cells were infected with serially diluted pseudoviruses, and the virus titer (ffu/mL) was measured by counting GFP-positive cells.

### Method details

#### Immunoblotting and PNGase F treatment

Cells were lysed in lysis buffer (50 mM Tris, 250 mM NaCl, 3 mM EDTA, 10% Triton X-100, 0.5% NP-40, and 10% glycerol). Cell lysate (10–50 μg) or viral supernatant (equal volume) were subjected to immunoblotting. Primary antibodies used in the study were anti-SARS-CoV-2 S2 (Genetex, Hsinchu, Taiwan; GTX632604), anti-VSV-M (Absolute, Boston, MA), and anti-beta-actin (Genetex, Hsinchu, Taiwan). The RBD antiserum was collected from rabbits immunized with RBD recombinant protein purified from *E*. *coli*. To remove N-linked oligosaccharides, viral supernatants were treated with peptide N-glycosidase F (PNGase F; NEB, Ipswich, MA) at 37°C for 1 h and then subjected to immunoblotting.

#### Immunofluorescence assay

293T/17 cells were transfected with plasmid DNA to express full-length or mutant S protein on coverslips, and the cells were fixed in 4% paraformaldehyde and permeabilized in 0.1% Triton X-100 of PBS buffer at 24 h post-transfection. Primary antibodies used in the study were: Spike S1 (Genetex; GTX635672), Spike S2 (clone 1A9, Genetex, GTX632604), mature S2 (clone ECD45, Antaimmu BioMed, Hsinchu, Taiwan; ATBM-A-08), PDI (Cell signaling, Danvers, MA), RCAS1 (Cell Signaling). Fluorescent secondary antibodies were purchased from Genetex or ThermoFisher. The cells were counterstained with DAPI (ThermoFisher) and examined by a confocal microscope (Leica TCS SP5).

#### Flow cytometry

The surface and intracellular staining of S protein for flow cytometry was performed with the protocol modified from the literature (Carter-Timofte et al., 2021; Cattin-Ortola et al., 2021; Chiuppesi et al., 2020). Briefly, HEK293T/17 cells were transfected with plasmid DNA to induce S protein expression for 24 h and dissociated from the culture dish with Versene solution (Gibco, Waltham, MA). For surface staining, cells were washed twice in FACS buffer (0.5% FBS in PBS), incubated with anti-mature S2 (1:500, ECD45) antibodies in FACS buffer for 30 min at 4°C, and staining with an Alexa 488-labeled secondary antibody for 30 min at 4°C. For intracellular staining, cells were fixed and permeabilized with Fix/Perm buffer (BD biosciences; ThermoFisher) for 5 min and washed three times in Perm wash buffer. The cells were then stained with anti-spike S2 (1:1000, clone 1A9) and fluorescent secondary antibodies for 30 min at 4°C. The stained cells were analyzed by flow cytometry (BD FACScalibur).

#### S protein-mediated cell-cell fusion assay

Calu-3 cells were seeded in a 12-well plate (1 × 10^6^ cells/well) and incubated for 48 h to form cell films. BHK-21 cells were co-transfected with Spike and GFP plasmids (5:1) for 24 h. The Spike/GFP expressing BHK-21 cells were harvested and added into the Calu-3 cell film at 4°C for 45 min. After PBS washing twice, the mixed cells were incubated in a D-MEM medium for cell-cell fusion at 37°C for 4 h. The fusion level was evaluated by examining GFP-expressing cells with an inverted fluorescence microscope (Olympus IX73). The GFP-expressing cell images were used to quantify cell size using the ImageJ software.

#### Modified acyl-PEG exchange (APE) to monitor S protein palmitoylation

S protein palmitoylation was detected by a modified version of the acyl-PEG exchange method described previously (Percher et al., 2016, 2017). HEK293T/17 cells expressing the wild type and mutant S glycoproteins were washed thrice in ice-cold PBS and cell pellets were stored at −80°C until lysis. Cells were lysed in 4SB buffer [4% (w/v) SDS, 150 mM NaCl, 50 mM triethanolamine pH 7.4] containing 1× Halt™ protease inhibitor cocktail (Thermo Scientific 78437), 5 mM phenylmethylsulfonyl fluoride (Sigma 329-98-6) and 1 μL/mL SuperNuclease (SinoBiological SSNP01). Protein concentrations were measured using the BCA assay (Thermo Scientific 23225). 1.5 μL of 0.5 M EDTA pH 8 (final EDTA concentration 5 mM) was added to 200 μg of protein in 141.75 μL lysis buffer prior to treatment with 10 mM TCEP (3 μL 500 mM TCEP, Thermo Scientific 77720) for 30 min at room temperature with rocking. 3.75 μL of freshly prepared 1 M N-ethylmaleimide (NEM, Sigma 04259, final concentration 25 mM) in ethanol was added to the samples and further incubated at room temperature for 2 h. Since we observed significant degradation for the S protein with the multiple precipitation steps in the APE protocol described by Percher et al., we chose to terminate reductive alkylation and remove excess NEM from the samples by buffer exchange using the Microcon-10 kDa filter units (Millipore MRCPRT010). Samples were concentrated and washed thrice with 500 μL SDS-TEA buffer (2% SDS, 150 mM NaCl, 50 mM triethanolamine pH 7.4, 4 mM EDTA) by centrifuging the tubes at 14,000 g for 1.5 h after each addition of SDS-TEA buffer. Samples (∼150 μL) were recovered by inverting the filter device over a new tube and centrifuging at 1,000 g for 5 min. Each sample was then split equally into two tubes (30 μL each). One half of each sample was subjected to hydroxylamine cleavage by adding 90 μL of freshly prepared 1 M NH_2_OH (Sigma 5470-11-1) in TEA buffer/Triton X-100 buffer pH 7.4 (final NH_2_OH concentration 0.75 M). As controls, 90 μL of TEA buffer/Triton X-100 buffer pH 7.4 was added to the other half of each sample. After incubation for 1 h with mixing at room temperature, samples were chloroform-methanol precipitated to remove the hydroxylamine by sequential addition of 4, 1.5 and 3 volumes of ice-cold methanol, chloroform and water, respectively. After mixing and centrifuging for 5 min at 20,000 g, 4°C, the upper aqueous layer was removed and 4 volumes of chilled methanol was added prior to mixing by gentle inversion. Proteins were pelleted by centrifuging for 2 min at 20,000 g, 4°C, washed with 1 mL chilled methanol, centrifuged again and air-dried prior to resuspension in 30 μL of 4SB buffer containing 4 mM EDTA. Sonication may be necessary to fully resuspend the proteins. For mPEG alkylation, 90 μL of freshly prepared 1.33 mM 10 kDa mPEG-Mal (Sigma 99126-64-4, final concentration 1 mM) in TEA/Triton X-100 buffer was added to each tube and incubated for 2 h with mixing at room temperature. Samples were then methanol-chloroform precipitated as described above. Air-dried protein pellets were resuspended in 50 μL SDS buffer before the addition of 17.4 μL 4× SDS-loading buffer (20% glycerol, 125 mM Tris·HCl, pH 6.8, 4% SDS, 0.05% bromophenol blue) and 2.6 μL TCEP (Thermo Scientific, 77720). Samples were heated for 5 min at 95°C, separated by SDS-PAGE, and transferred to PVDF membranes for Western blot analysis using the anti-S antibody (Genetex GTX632604) and secondary antibody (Jackson ImmunoResearch 115-035-003).

#### Cell proliferation assay

Cell proliferation kit was following the manufacturer’s instructions and previously described methods (Tsai et al., 2016). In brief, 1×10^4^ of 293T cells were cultured in 96-well plates. After 2BP treatment at 37°C for 24 h, the MTT labeling reagent (Sigma-Aldrich, Darmstadt, Germany) was added to each well at a final concentration of 0.5 mg/mL for additional 4 h incubation. Finally, the reaction was terminated by adding the stop solution, and the absorbance was measured at 570 nm.

### Quantification and statistical analysis

All data are represented as the mean ± SEM and were analyzed using one-way ANOVA with Tukey’s test. A p-value < 0.05 indicates a statistically significant difference.

## Data Availability

• Data reported in this paper will be shared by the lead contact upon request. • There is no original code associated with this work. • Any additional information required to reanalyze the data reported in this paper is available from the lead contact upon request.
