# Supplementary material for: Glycosylation and S-palmitoylation regulate SARS-CoV-2 spike protein intracellular trafficking
Source: iScience. 2022 Jul 3;25(8):104709. doi: 10.1016/j.isci.2022.104709 (PMC9250814; doi:10.1016/j.isci.2022.104709)
Supplement: Document S1. Figures S1–S9 [file mmc1.pdf]

iScience, Volume *25*

Supplemental information

Glycosylation and S-palmitoylation regulate SARS-CoV-2 spike protein intracellular trafficking

Chih-Feng Tien, Wan-Ting Tsai, Chun Hwa Chen, Hui-Ju Chou, Mingzi M. Zhang, Jhe-Jhih Lin, En-Ju Lin, Shih-Syong Dai, Yueh-Hsin Ping, Chia-Yi Yu, Yi-Ping Kuo, Wei-Hsiang Tsai, Hsin-Wei Chen, and Guann-Yi Yu

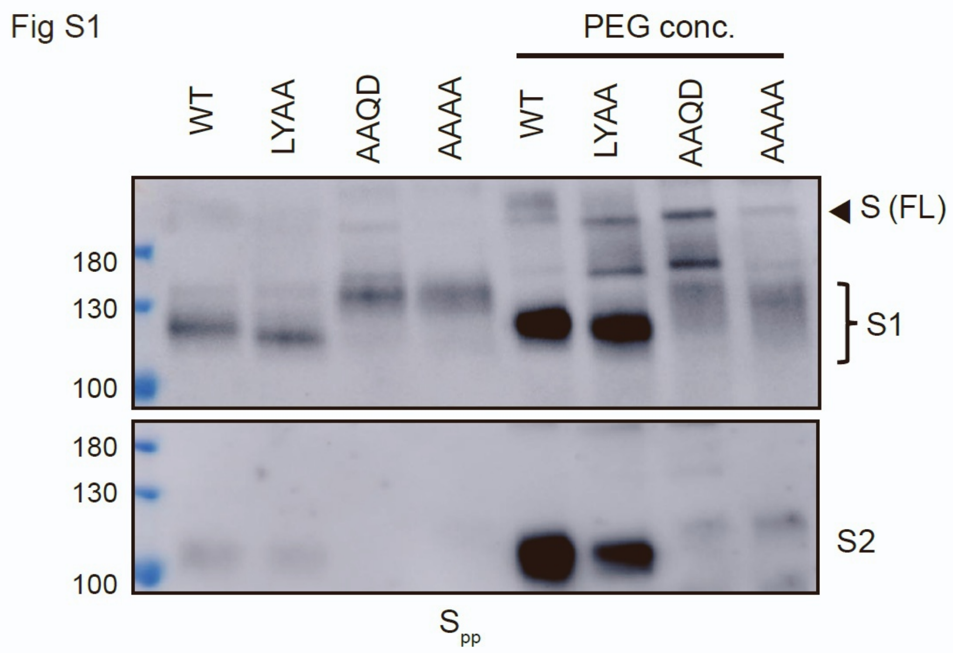

Figure S1. S_{pp} and the PEG-precipitated S_{pp} from 293T cells were detected by immunoblotting, related to Figure 1.

Fig S2

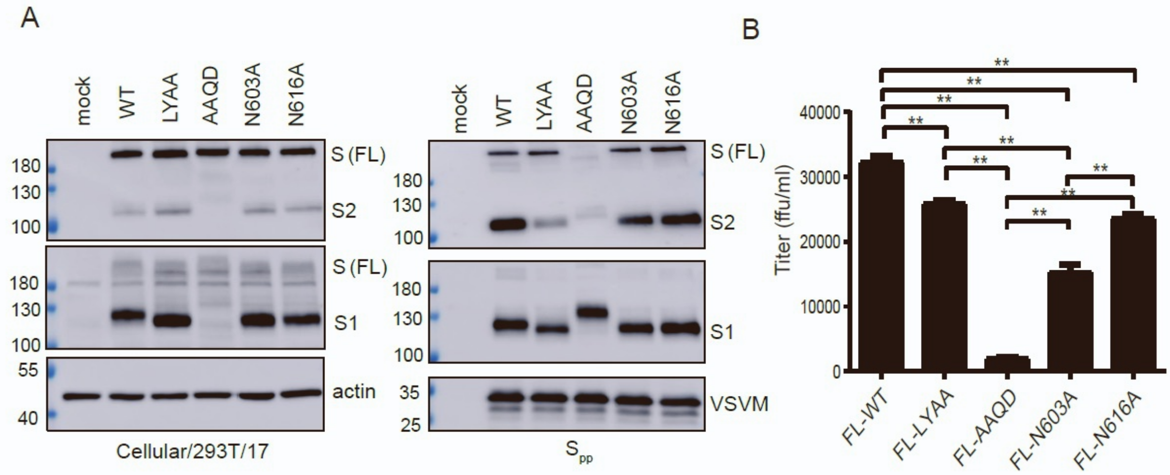

Figure S2. S protein with a N603A or N616A mutation was used for pseudovirus production, related to Figure 1. Protein expression (A) and S_{pp} titer (B) were examined. Error bars represent SEM.

Fig S3

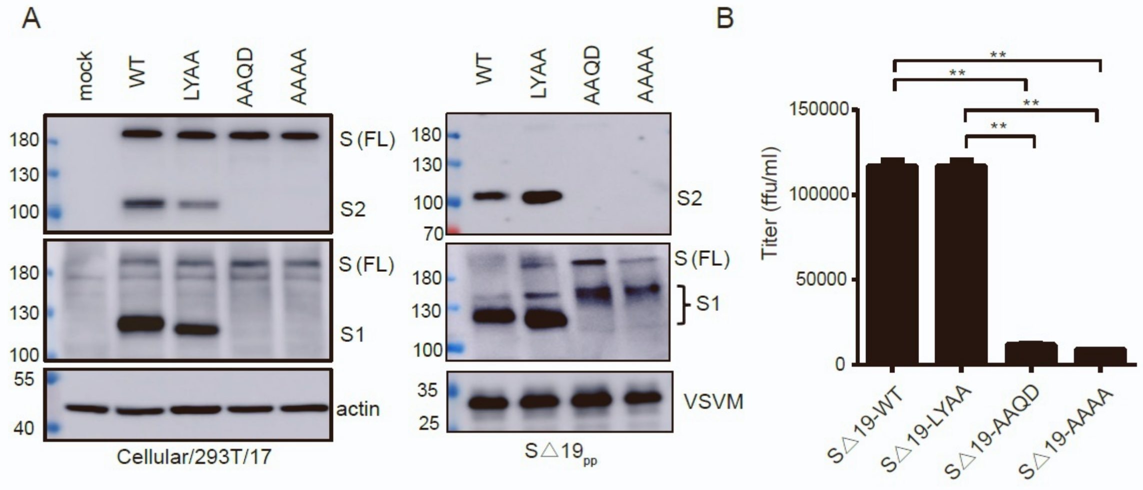

Figure S3. S protein with a 19 amino acid deletion at the C-terminal tail (SΔ19) was used for pseudovirus production (SΔ19$_{pp}$), related to Figure 1. Protein expression (A) and SΔ19$_{pp}$ titer (B) were examined. Error bars represent SEM and n=3.

Fig S4

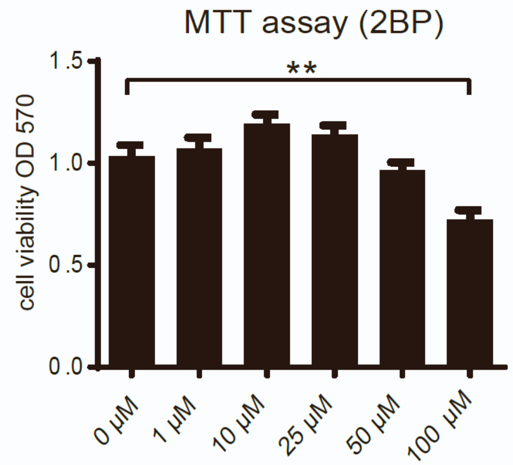

Figure S4. The cytotoxicity of 2BP treatment, related to Figure 3. 293T/17 cells were treated with 1-100 µM of 2BP for 24 h, and the cell viability was evaluated by the cell proliferation assay. **P<0.01. Error bars represent SEM.

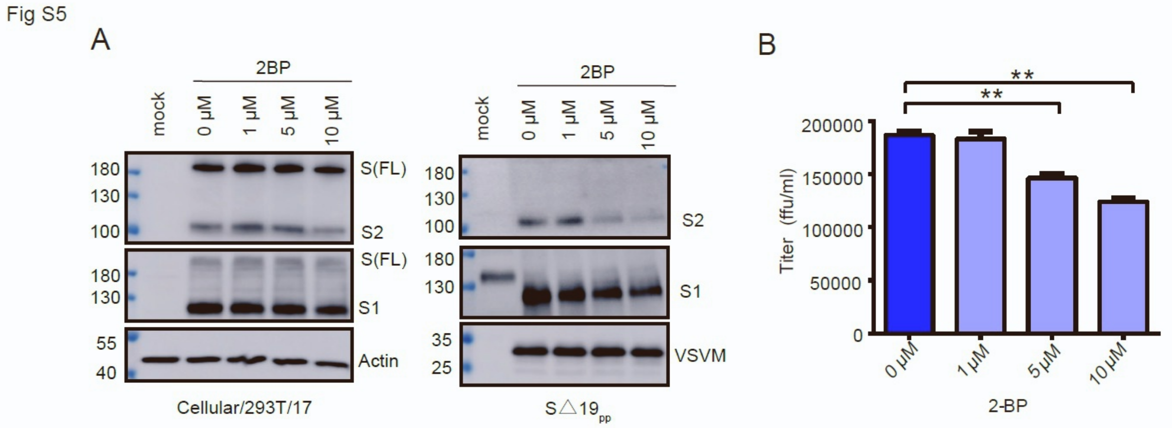

Figure S5. The effect of 2BP to SΔ19$_{pp}$ production, related to Figure 3. (A) SΔ19 protein expression in 293T/17 cells and SΔ19$_{pp}$ were detected by immunoblotting. (B) The SΔ19$_{pp}$ titer in the culture medium was measured. Error bars represent SEM and n=3.

Fig S6

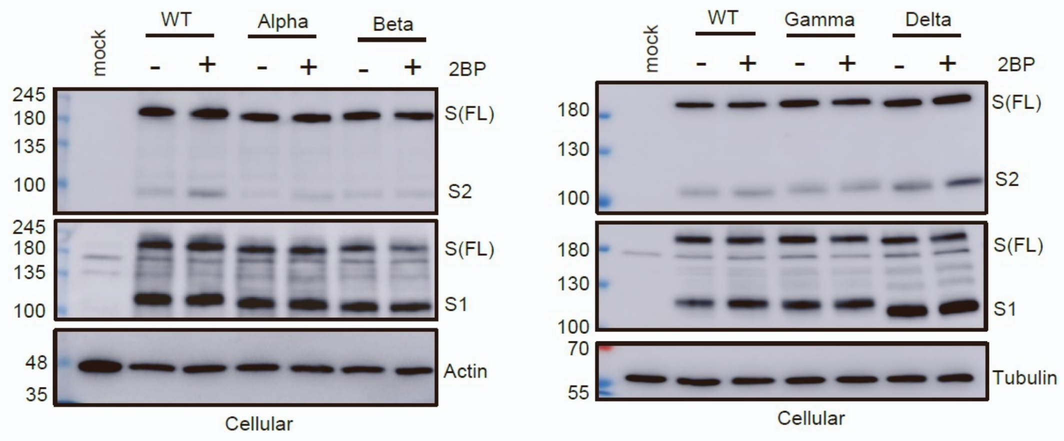

Figure S6. The WT and variant S proteins (alpha, beta, gamma, and delta strains) expression in 293T/17 cells in the presence of 2BP (10 μM) was examined by immunoblotting, related to Figure 3.

Fig S7

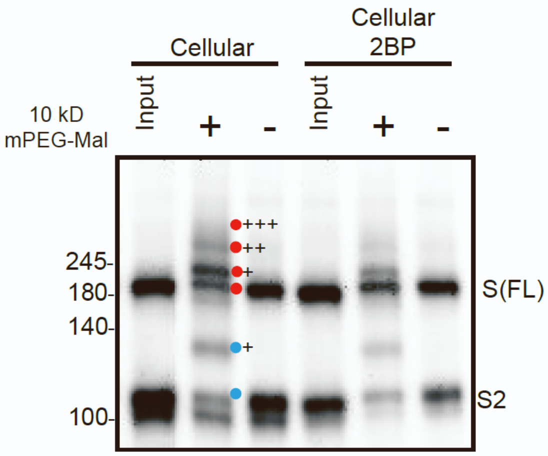

Figure S7. Palmitoylation level of the SΔ19 protein with or without 2BP, related to Figure 4. The SΔ19 protein in 293T/17 cells was treated with 10 kDa mPEG-mal and monitored by immunoblotting with anti-S2 antibody. Red circle: SΔ19 FL protein; blue circle: SΔ19 S2 subunit. +: number of different palmitoylated species.

Fig S8

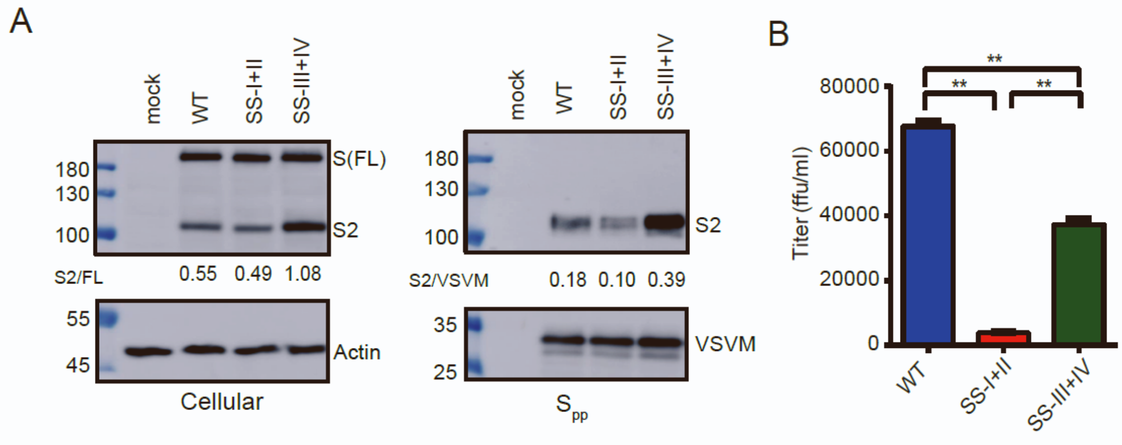

Figure S8. The effect of cysteine clusters I+II and III+IV combination mutations in S protein processing and S_{pp} production, related to Figure 4. (A) The S protein expression of WT and cysteine mutants (I+II and III+IV) in 293T/17 cells and S_{pp} was examined by immunoblotting with an anti-S2 antibody. (B) The titers for WT and cysteine mutants S_{pp} were evaluated. **P<0.01. Error bars represent SEM and n=3.

Fig S9

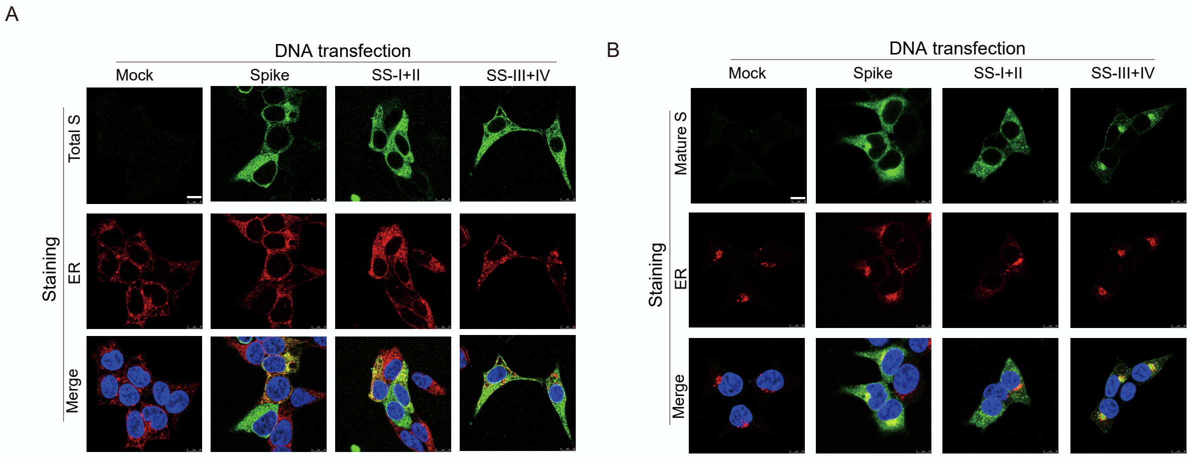

Figure S9. Localization of the S protein with cysteine-rich cluster I+II and III+IV mutations, related to Figure 5. The S protein expression of WT and cysteine mutants (I+II and III+IV) in 293T/17 cells for 24 h was double-stained with antibodies for S1 (nascent S) and PDI (ER marker) (A) or S2 (ECD45 for mature S) and RCAS1 (Golgi marker) (B). Scale bars: 10 μm.
